# Effect of Ambient Oxygen Content, Safety Shoe Type, and Lifting Frequency on Subject’s MAWL and Physiological Responses

**DOI:** 10.3390/ijerph16214172

**Published:** 2019-10-29

**Authors:** Atef M. Ghaleb, Mohamed Z. Ramadan, Ahmed Badwelan, Khalid Saad Aljaloud

**Affiliations:** 1 Department of Industrial Engineering, College of Engineering, King Saud University, Riyadh 11421, Saudi Arabia; mramadan1@ksu.edu.sa (M.Z.R.); A.BADWELAN@gmail.com (A.B.); 2 Department of Exercise Physiology, College of Sport Sciences & Physical Activity, King Saud University, Riyadh 11451, Saudi Arabia; khaljaloud@ksu.edu.sa

**Keywords:** ambient oxygen content, EMG, lifting, manual materials handling, physiological responses, maximum acceptable weights lifting

## Abstract

Objective: The purpose of this study was to evaluate the lifting capabilities of individuals in hypoxia when they wear different types of safety shoes and to investigate the behavior of the physiological responses induced by the lifting process associated with those variables. Methods: An experimental design was used, based on two sessions. The first was training and acclimatization session, then an experimental lifting phase. A total of ten male students of King Saud University were recruited in the study. A four-way repeated measures design, with four independent variables and six dependent variables, was used in this research. The independent variables that were studied in the experimental lifting phase were: ambient oxygen content (15%, 18%, and 21%), safety shoes type (light-duty, medium-duty, and heavy-duty), lifting frequency (1 and 4 lifts/min), and replication (first and second trials). The dependent variables were also: maximum acceptable weights lifting using the psychophysical technique, heart rate (HR), electromyography (EMG) of (biceps brachii, trapezius, anterior deltoid, and erector spinae), safety shoes discomfort rating, rating of perceived exertion, and ambient oxygen discomfort rating. Results: The maximum acceptable weights lifting that were selected by participants at lower levels of the independent variables (ambient oxygen content 21%, lifting frequency 1 lift/min, and first replication) were significantly higher than at high levels of the independent variables (ambient oxygen content 15%, lifting frequency 4 lift/min, and second replication). Several interaction effects were also significant. Conclusions: It provides evidence that the ambient oxygen content increases the intensity of workload in lifting tasks. It showed that oxygen content affects the psychophysical selection of maximum acceptable weights lifting and the physiological responses represented in muscular activities and heart rate. It suggests that ambient oxygen content must be considered along with the type of safety shoes worn when the lifting task at altitudes occurs.

## 1. Introduction

Over the years, there is an increase in the studies concerning the manual material handling task. Mirta [1] resonates that this is because of the increased documentation of work-related musculoskeletal disorders (WMSDs). Similarly, Mavor and Graham [2] have demonstrated growing episodes of workplace injuries across all occupations, gender, and age. Generally, manual task handling and lifting occur frequently in both occupation and non-occupational jobs, as per its specific requirement, significantly contributing to the increased lower back pain [3]. The occurrence of lower back pain and related musculoskeletal disorders triggered by work rank as second common reasons for referral to physicians following cardiovascular diseases [4].

A recent study by Shojaei et al. [5] illustrated that work-related musculoskeletal disorder impacts the quality of the industrial workers, and affects the overall production capacity of the company, globally. Vos et al. [6] concluded that lower back pain has accounted for worker’s disability across 139 countries from a total of 188 countries. Not only this, but low back pain (LBP) also adds to the economic and medical burden of the nation, due to lost time, and decreased productivity [7]. It was found in the Workplace Safety and Insurance Board (WSIB) statistical report [8] that a total of 7.7 days of work are lost in a month due to a workplace injury, which accounted for an average of 14.1 working days within three months. Most studies have emphasized on understanding the critical mechanistic and environmental factors that add to the global prevalence and development of LBP [2,5,6].

Evidence from the literature has drawn attention to several parameters (frequency, work postures, load, vertical and horizontal distance, etc.) that impact the occupational workers [1,9,10]. Such as, an earlier study of Ciriello and Snook [11] assessed the influence of frequency, distance, height, and size of the box on the worker concerning his lifting, pulling, and pushing tasks by measuring oxygen consumption and heart rate(HR). The study concluded that frequency, distance, height, and size important variables should be considered when determining maximum acceptable weights (MAW). Similar results are indicated by the recent research of Abadi et al. [12] on Iranian workers, which reported that variables such as lifting frequency and the box size affected worker’s maximum acceptable weight of lift (MAWL) and heart rate.

Analysis of experimental studies has shown that generally, the focus of the lifting tasks for the previous researches has been centered on a single parameter rather than all [9,10,12]. Moreover, those studies, which have included all parameters, were restricted to the Western work population such as America, Australia, Britain, and more [13,14,15]. In order to expand the research area further, the study evaluates the materials handling activities in Saudi Arabia constituting, all parameters of lifting tasks as an integrated group. Many researchers had investigated a person’s capacity to do manual handling activities in a normal environment or a hot environment. Despite it, the influence of different hypoxia levels on manual handling activities is found to be an untested area. The lack of literature concerning the safety guidelines regarding worker’s lifting capabilities while wearing safety shoes also encourages the researcher to carry out this study on the Saudi population. 

Moreover, Ayoub [16] provided three methods to control the lifting risk, which can be used separately or together. These include; (a) hazard control through training in safe lifting, (b) control of employee exposure through pre-employment strength testing, and (c) control of job demands through the adoption of workloads acceptable for the industrial worker. Snook et al. [17] studied 191 cases of lower back (LB) injuries to define the effectiveness of prophylactic methods utilized in industrial establishments. They have noticed that the popular selection approaches were not an effective control for injuries of LB, as well as training on procedures of safe lifting. The authors noted that the worker who performed extensive manual handling tasks is three times more likely to experience back pain.

Similarly, Maiti [18] proposed a technique to define the mean steady pause time from fluctuating working heart rate and frequency of lifting. This kind of load-handling task presented a higher relative heart rate and lower work efficiency. Abadi et al. [3] supply that this pain increases the absenteeism of the worker, impacting the overall functionality of the nation.

Many researchers evaluated different factors during manual material handling activities during different conditions. They were working heart rate based on the maximum heart rate obtained through maximum aerobic power measurement [19], heart rate, oxygen consumption, and rating of perceived exertion (RPE) [20,21], the pressure of blood [21], and an increased hazard of lower back pain (LBP) in different professional settings [22]. It was also considered influences of the width of the container, distance, and rate of carrying on the HR, maximum acceptable weight carried (MAWC), and RPE through a one-hour carrying task [23]. Impacts of six various lifting variables; horizontal distance, load weight, lifting frequency, duration of work, lifting height and twisting angle, and prophesy the lifting effort and the risk of injury through a huge configuration lifting [24], the impacts on muscles using electromyography (EMG) through asymmetric lift from the ground [25], psychosocial hazard detection and musculoskeletal disorders (MSDs) across women and men of working age [26], and the effects of ambient temperature on physiological responses during manual lifting tasks [27,28].

The amount of oxygen in the air indicates molecules of oxygen present in the air per volume unit that decreases as the altitude increases. Furthermore, the pressure of atmospheric significantly affects the functions of the human body because low atmospheric pressure leads to a decrease in partial oxygen pressure [29]. Thus, the change in atmospheric pressure and the molecular pressure of oxygen are vital variables affecting oxygen transport and human respiration in altitude [30]. Many studies [31,32] have shown that the maximal workload and uptake of oxygen are directly related to the atmospheric pressure, and the partial pressure of oxygen, which decreases in the variables, causes a decrease in other (in other words, when altitude increases).

If the amount of oxygen reaching the cells is insufficient, the hydrogen interacts with the pyruvic acid and converts it into lactic acid. This temporary anaerobic metabolism produces a small amount of energy. The accumulation of lactic acid in the blood and tissue indicates that there is an insufficient amount of oxygen in the mitochondria, which can be due to hypoxia or lack of blood flow (such as shock) or a mixture between them [33]. If this is prolonged or severe, it can lead to cell death. Moreover, during activity, performance is determined by the amount of oxygen transferred by the circulatory system to the muscles [34]. Muscle fatigue occurs when the intensity of the activity is high due to a lack of oxygen enough to get the metabolism and the accumulation of inorganic phosphate [35].

Sudden exposure to high altitude leads to increased heart rate as well as ventilation, “mainly driven by hypoxemia-induced carotid chemoreceptor activation, sympathoexcitation and vagal withdrawal” [36,37], as the increase in heart rate compensates for the lack of oxygen. When a person stays in high places for several days, resting heart output normalizes because of the lowering in the volume of stroke due to the decrease of plasma volume because of the hypoxia [38]. However, after acclimatization, the heart rate and ventilation continue to increase by “a sustained sympathetic response driven by peripheral chemoreceptors sensitized by the persistent hypoxemia” [39,40]. 

There are also few studies on the effect of shoes during the lift tasks. Aghazadeh and Lu [41] studied the impact of changing the position of the body that result by wearing high heel shoes “flat, 5 and 7.6 cm” during the lifting “(F-K) floor to knuckle and (K-S) knuckle to shoulder” on maximum lifting capacity. This research displays that high heel shoes might have an impact on lifting and back injuries. Li et al. [42] studied the influence of footwear–floor slipperiness conditions for material handling workers who work in various footwear–floor slipperiness conditions. They conclude that there is a significant effect for friction level on a perceived sense of slip, the MAWH, Vo2, and efficiency of energy. Kim et al. [43] examined the kinematics of the lower extremity and trunk and characteristics of EMG in the sit-to-stand (STS) job when wearing deferent high-heeled shoes (1, 4, and 8 cm). The results show that there is a significant difference in EMG activity through the deferent high-heeled STS conditions.

Al-Ashaik et al. [28] assessed the lifting capabilities when wearing various safety shoe types in a warm environment and examined the physiological responses during lifting tasks. They concluded, “The heat stress increases the workload intensity in manual lifting tasks influencing the psychophysical selection of MAWL and the physiological responses of the human body represented in aural-canal temperature, heart rate, and muscular activities. The study findings demonstrated the necessity of accounting for work environmental temperature and type of worn safety shoes, which is a safety requirement by most employers when calculating the recommended weight limits”. The scarcity of research concerning the integrated impact of the discussed variables; the present study aimed to assess the lifting capabilities of the individuals in hypoxia when wearing different types of safety shoes. It also examines the physiological responses produced by the lifting tasks associated with these factors. Moreover, this psychophysical study was based on the hypothesis; such as, the oxygen content, worn safety shoes, lifting frequency, and replication have a significant effect on (i) MAWL (ii) HR (beats per min), (iii) EMG of four muscle groups “trapezius, erector spinae, anterior deltoid and biceps brachii”, (iv) rating of perceived exertion, (v) safety shoes discomfort rating, and (vi) ambient oxygen discomfort rating. The current study differs from previous researches based on its study objective, participants, region, and scope.

## 2. Materials and Methods

### 2.1. Study Design

The study used the experimental study design for achieving the determined objectives, integrating into a quantitative approach. The rationale for selecting this particular study design is twofold, such as it is found to provide results in an easy to comprehend form and that too statistically [44]. Secondly, this research design has been effective in deriving concrete and complete results for the other similar researches [2].

### 2.2. Study Participates

Ten healthy male students from King Saud University with a mean age (standard deviation) of 26.3 (2.53) years were recruited in this study, as per the determined inclusion and exclusion criteria (Table 1). Participants’ mean weight 66.63 (8.04) kg, mean height 165.1 (3.01) cm, mean shoulder height 138.7 (2.9) cm, mean hip height 85.4 (3.29) cm, mean knuckle height 67.55 (2.03) cm, mean knee height 48 (2.19) cm, and mean arm length 63.4 (2.46) cm. The followings were the inclusion criteria for this study: (1) participants who have not experienced any back or lower and upper limb problems; (2) participants who have not experienced heart disease; and (3) participants who have not experienced breathing problems.

### 2.3. Ethical Consideration

Prior to the study, ethical clearance was obtained from the Institutional Review Board at King Saud University (ethical code # E-19-3752). This process was done by submitting a written proposal inclusive of all the study details; scope, objective, methods, participant’s requirement, and protocols. Moreover, a written consent form was also obtained from the students after communicating them explaining the study objective, confidentiality as well as anonymity of the data. The researcher also communicated the participant’s right to withdraw at any time of the research.

### 2.4. Equipment

All experiments were performed inside the environmental chamber (6 m × 6 m × 3 m), made in (Weiss Technik UK LTD, Loughborough, UK). The environmental chamber is self-controlled of air content concerning the percent of oxygen content, dry-bulb temperature, and relative humidity inside the chamber, and presented all concerned data in a display. In addition, a SENSIT^®^ P400 Multi-Gas Monitor was used to gauge the ambient oxygen content before collecting data at any session. An eight-channel Biomonitor ME6000, MT-ECG-1 preamplifier, and Mega Win 3.0.1 software (Mega Electronics Ltd., Kuopio, Finland) were used to record the physiological signals, and two-handle box (40 cm × 60 cm × 22 cm) was used for lifting the loads. Headphones were used to reduce the noise inside the chamber and three types of safety shoes were used in the experiment manufactured by Shelterall Company, Italy. In addition, the Kubios HRV software v2.2 (University of Eastern Finland, Kuopio, Finland) was used to compute the heart rate. Other materials and equipment included 70% isopropyl-alcohol swaps, cotton squares, Band-Aid, Ag/AgCl solid adhesive pre-gelled electrodes for heart rate, and EMG signal acquisition (Ambu A/S, Ballerup, Denmark) and high-viscosity electrolyte gel for active electrodes. The eight-channel Biomonitor ME6000 unit was calibrated based on the manufacture’s recommendations.

### 2.5. Experimental Variables

**Figure 1:**
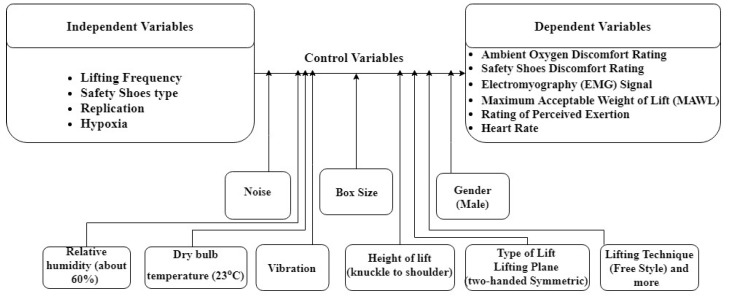
Study variables.

The independent and dependent variables of the experiment were as follows (Figure 1).

#### 2.5.1. Independent Variables

• Hypoxia:

This study performed under three conditions of oxygen content environments, i.e., 15%, 18%, and 21% O_2_ content in the air. The selection of these environments was based on the altitude of different cities on the sea level.

• Lifting frequency:

The selection of the lifting frequency as the independent variable was based on its inclusion in the existing studies. Similar to most of the psychophysical studies conducted in the lifting used the lifting frequency as an independent variable. They concluded that MAWL decreases with increasing lifting frequency, while also establishing a linear relationship between energy expenditure and lifting [45]. The frequencies used in this study were 1 and 4 lifts/min [46,47].

• Safety shoe type:

Few studies that evaluate the effect of shoes during the lifting tasks concluded that leg and back stresses are affected based on the different types of shoe. Thus, in this study, three types of safety shoes, i.e., light-duty, medium-duty, and heavy-duty, were used, similar to those in [28]. There were three types of shoes, and each one was in different sizes so that each participant wore the size that suits his foot. The definition of the shoe type comes from manufacturing. For more details about the safety shoe type, please see study [28].

• Replication:

This study performed with two replications based on the difference in the weight in the start and following the experiment. Such as the first replication was in the start with 30% of 1RM, while in the other, it started from the no weight, and it was random for every session.

#### 2.5.2. Dependent Variables

• Maximum acceptable weight of lift (MAWL):

It is a well-known fact that as the load lifted increases, the mechanical work needed to lift it also increases, which in turn increases the amount of energy expended by the worker. Mital [48] have shown that an increase in the load to be lifted leads to an increase in the metabolic energy rate. Therefore, the participants randomly started with the weight initially (no weight or 30% 1RM) and then asked the helper to increase or decrease the weight until the weight reaches the appropriate weight (maximum acceptable weight), which the participant can lift it for 8 h without straining himself. The participants were given 15 min to determine the maximum acceptable weight and then continued to lift the weight that was determined for 5 min without any adjustments.

• Heart rate:

Many researchers have shown that heart rate is directly proportional to workload, lift frequency, and heat stress [28,46,49,50]. The heart rates of the participants were measured using “an eight-channel Biomonitor ME6000 (Mega Electronics Ltd., Kuopio, Finland)”. The signals were recorded using “Mega Win 3.0.1 (Mega Electronics Ltd., Kuopio, Finland)” software. Additionally, the heart rate was calculated using Kubios HRV software v2.2 (University of Eastern Finland, Kuopio, Finland).

• Electromyography (EMG) signal:

EMG explains the schematic technique of muscle action potentials with surface electrodes and examines their features [51]. In this study, surface EMG was collected using the ME6000 system and Ag/AgCl surface electrodes from four muscle groups “trapezius, erector spinae, anterior deltoid, and biceps brachii” [28,52,53].

• Rating of perceived exertion:

The rating of perceived exertion (RPE) scale was used to measure the intensity of work being performed. Borg developed the RPE; the RPE scale runs from 6–20 [54].

• Safety shoes discomfort rating:

This measure reflected the workers’ sense of discomfort while wearing safety shoes. The participants assessed the shoes based on Table 1 below. Participants in their experiment were asked to rank their discomfort sensation due to wearing safety shoes, orally at the end of each session. Instructions for rating safety shoes discomfort were given to the participants. It was also posted on the chamber wall in front of the participant during the experimental session.

• Ambient oxygen discomfort rating:

This measure reflected the workers’ sense of ambient oxygen during the study. Participants in their experiment were asked to rank their discomfort sensation due to difficulty of breathing, orally at the end of each session. Instructions for ambient oxygen discomfort ratings were given to the participants. It was also posted on the chamber wall in front of the participant during the experimental session.

#### 2.5.3. Control Variables

The controlled variables contained all the variables, which were kept constant during the study. In this study, all other variables like dry bulb temperature (23 °C), relative humidity (about 60%), vibration, noise, the height of lift (knuckle to shoulder), size of the box, gender (male), type of lift lifting plane (two-handed symmetric) and lifting technique (freestyle), etc., were considered as controlled.

### 2.6. Experimental Procedures

The protocol that was followed in this study was categorized into two phases. The first phase was concentrated on the training and acclimatization sessions, whereas, the second phase constitutes experimental lifting. Following attainment of the consent form from the participants, the anthropometric data (such as body weight, shoulder height, hip height, knee height, arm length, knuckle height, and body height) as well as the maximum amount of weightlift in one lift (1RM) were measured and recorded. Based on this measurement, the participation schedule was issued to the participants.

#### 2.6.1. Training and Acclimatization Sessions

Training is necessary for participants to develop their muscles and lifting capabilities so that they become more representative of workers in the industrial environment. Training also reduces the learning effect later when conducting the experimental lifting phase. Regarding the training and acclimatization, the study was for two hours each day for 14 consecutive days [55]. In it, the participants were initially trained on the content of oxygen, i.e., 18% for the first day to become more used to it for the experiment and then trained on the content of oxygen 15% for the next thirteen sessions. During the training and acclimatization program, each participant was also asked randomly and sequentially to wear one pair of safety shoes that was to be assigned in the experiment. The intended program to train the participants for the following: flexibility, muscular strength, and muscular endurance was similar to the procedures employed by Ramadan [49].

#### 2.6.2. Experimental Sessions

Once the participants were acclimatized and ready for the new environment, the procedure of data collection began. Each participant was asked to lift a weight from knuckle to shoulder. Each participant lifted the weight under each of the 18 experimental conditions in 18 days (one experimental condition a day), because there were three independent variables: ambient oxygen content ((21%, 18%, and 15%; these were equivalent to attitude 0, 1500, and 2760 m above sea level, and these altitudes represent low, moderate, and high altitude respectively) [56]. Nearly 98% of the world’s population also lives at 2500 m or less [57]), lifting frequency (1 and 4 lift/min) [47], and safety shoes (light-duty, medium-duty, and heavy-duty) [28]. In addition, before the experiment, participants were asked to sleep well and enough and stay away from smoking, caffeine, and tea for at least four hours prior to the experiment.

Previous to conducting the lifting task, every participant was equipped with surface electrodes of EMG, which were placed on the four muscles (trapezius, erector spinae, anterior deltoid, and biceps brachii) by the same physician. Due to the critical situation of executing such type of experiment, the Sport Sciences and Physical Activity department assigned on-site physician to take care of the participants’ medical needs as well as EMG electrodes positioning. Standard steps for placing the electrodes of EMG were followed [58]. Afterward, maximum voluntary contraction (MVC) measured and recorded (every MVC test was for 5 s) to be utilized as a reference to mirror the proportion of muscle contraction performance capacity [59]. An intense, continuous, and loud oral spur was done via the study team to decrease the limitation of muscle contraction capacity by the insufficiency of inhibitory and motivation effects [60]. Three experiments were done with a three minutes rest in between to eliminate the fatigue of the muscle. The MVC was measured and recorded prior to every session to be used as a covariate of the EMG data of its corresponding session (to remove the influences of changed testing day and different locations of the electrode).

After that, the surface heart rate electrodes were placed on the chest of the participant. Following it, every participant performed the lifting task within the chamber for every 18 conditions of the experiment. The lifting was from knuckle to shoulder in the sagittal plane with no twisting using the freestyle method. Participants lifted a two-handle box (40 cm × 60 cm × 22 cm) having weights inside. After lifting the box to the shoulder level, an assistant would align the box to the knuckle level. Five minutes pre-work-rest was allowed to each participant, followed by fifteen minutes of lifting psychophysically to determine his MAWL [49]. After a participant affirmed his MAWL for the experimental session, he continued lifting by this MAWL for the next five minutes. Then, the participant was allowed ten minutes to rest and recover (within the chamber), after which the second replication of lifting for another twenty minutes occurred, similar to the first replication. 

Afterward, the second replication, the participant preordains five minutes inside the chamber to take a rest and recover. The difference between the two replications was in the starting weight, which was different from the second one. Such as, in one replication, it was to be started with 30% of 1RM, while in the other it started from no weight, which was done randomly for every session. In case the MAWL was determined in the same session within 10 percent, then it was considered for statistical analysis; otherwise, the third replication was performed.

The orders of the experimental sessions were randomized. The prescripts that were to be given to the participants was similar to that suggested by [61]; where the participants on their own selected the MAWL efficiently which they could lift under a particular condition for eight hours every workday “without straining themselves or without becoming unusually tired, weakened, overheated, or out of breath”. Numerous researchers [62,63,64,65,66,67,68,69] recommended that the psychophysical method is a credible technique to evaluate the perceived exertion through the manual materials handling (MMH) task in low and moderate frequencies (<6 lifts/min).

For every minute of the five minutes pre-work rest, the heart rate at rest was measured. For another five minutes, i.e., between the two replications and after the second replication, the recovered heart rate was also measured. Through the two replications of the five minutes of lifting using the MAWL, physiological responses (heart rate, and EMG signals) was measured. After each of the lifting replications, participant’s rating of perceived exertion (RPE), safety shoes discomfort rating, and rating of oxygen feeling (ROE)) were obtained. The only principal researcher performed all measurements on all participants on all test days.

### 2.7. Statistical Analysis

The analysis of the collected data was done using IBM SPSS (Statistical Package for Social Sciences) version 23.0. A four-way repeated measures design, with four independent variables and six dependent variables. Therefore, the experiment had 36 conditions based on the combinations of levels of the independent variables. The least significant difference method was used for pairwise comparisons of the main effects to identify significantly different levels of the main variables. In addition, if an interaction was found to have a significant effect on the dependent variables, a simple effect technique was conducted to demonstrate the effect at each level of the safety shoe type and ambient oxygen content factors [70]. The Shapiro–Wilk test was implemented to test data normality [71]. The statistical significance was set at a confidence level of 95%.

## 3. Results

### 3.1. MAWL

The results of the experiment showed that three main factors; ambient oxygen content (F (2.8) = 6.716, *p* < 0.019), lifting frequency (F (1.9) = 52.78, *p* < 0.001), and replication (F (1.9) = 7.773, *p* < 0.021)) had a significant effect on MAWL, as shown in Table 2. It showed that MAWL was significantly higher at ambient oxygen content of 21% (mean (SD) = 17.46 (3.62) kg) when compared to ambient oxygen content of 18% (mean (SD) = 17.01 (3.67) kg), or ambient oxygen content of 15% (mean (SD) = 15.97 (3.39) kg). Additionally, the MAWL was significantly less when lifting frequency was 4 lift/min (mean (SD) = 14.96 (3.73) kg) when compared to 1 lifts/min (mean (SD) = 18.66 (3.38) kg). Finally, The MAWL was significantly higher at first replication (mean (SD) = 16.89 (3.59) kg) when compared to second replication (mean (SD) = 16.74 (3.53) kg).

### 3.2. Heart Rate

**Figure 2:**
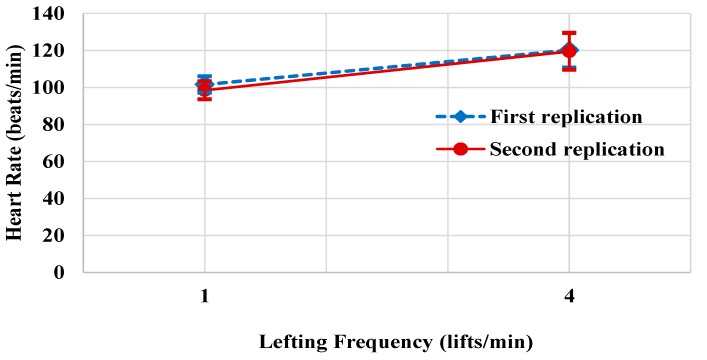
Effect of lifting frequency by replications on heart rate.

It was observed that two main variables; lifting frequency (F (1.9) = 30.028, *p* < 0.001) and replication (F (1.9) = 11.545, *p* < 0.008), as well as a two-way interaction between lifting frequency and replication, had a significant effect on the heart rate of participants, as shown in Table 3. The experiment reveals that heart rate was significantly higher when lifting frequency was 4 lift/min (mean (SD) = 119.79 (16.26) beats/min), when compared to 1 lifts/min (mean (SD) = 99.98 (10.52) beats/min). Moreover, the heart rate was significantly higher at first replication (mean (SD) = 110.85 (13.36) beats/min), when compared to second replication (mean (SD) = 108.92 (13.42) beats/min). As shown in Figure 2, at lifting frequency of 1 or 4 lifts/min, the heart rate was significantly higher at first replication when compared to second replication.

### 3.3. Electromyography (EMG)

#### 3.3.1. Biceps Brachii Muscle

The main factor (lifting frequency) had a significant effect on the normalized root mean square of Biceps brachii muscle (%MVC), F (1.9) = 129.22, *p* < 0.001. The %MVC of the brachii muscle with lifting frequency 4 lifts/min (mean (SD) = 8.84% (3.05)) was significantly higher than the %MVC when the lifting frequency 1 lift/min (mean (SD) = 3.494% (1.55)). Moreover, the two-way interaction between safety shoe type and lifting frequency has a significant effect on the normalized root mean square of Biceps brachii muscle (%MVC), F (2.8) = 4.764, *p* < 0.043.

**Figure 3:**
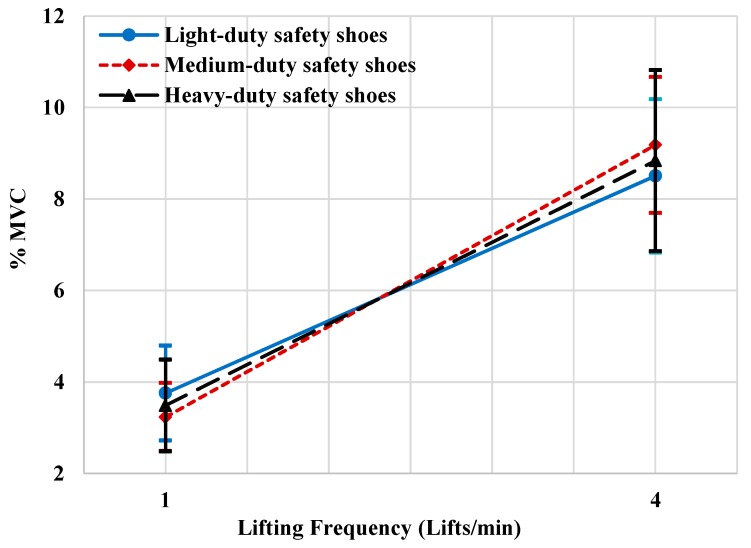
Effect of lifting frequency by safety shoe type on % maximum voluntary contraction (MVC) of biceps brachii muscle activities.

Figure 3 shows that at lifting frequency of 1 lift/min, the % MVC was significantly lower when wearing the medium safety shoes than when wearing light-duty safety shoes or-heavy-duty safety shoes. Moreover, the %MVC at Lifting frequency of 4 lifts/min was significantly lower when wearing the light-duty safety shoes than when wearing the medium-duty safety shoes or heavy-duty safety shoes.

#### 3.3.2. Anterior Deltoid Muscle

The lifting frequency only had a significant effect on the normalized root mean square of the anterior deltoid muscle (%MVC), F (1.9) = 101.636, *p* < 0.001. The normalized root means square of the anterior deltoid muscle (%MVC) at a lifting frequency of 4 lift/min was significantly higher (mean, SD = 9.781%, 3.228%) than at a lifting frequency of 1 lift/min (mean, SD = 3.741%, 1.308%).

#### 3.3.3. Trapezius Muscle

The lifting frequency, three-way interaction between ambient oxygen content, lifting frequency, and safety shoe type, and four-way interaction between ambient oxygen content, lifting frequency, safety shoes type, and replication had a significant effect on the normalized root mean square of trapezius muscle (%MVC), F (1.9) = 81.947, *p* < 0.001, F(4.6)= 5.290, *p* < 0.036, and F(4.6) = 5.091, *p* < 0.039, respectively.

**Figure 4:**
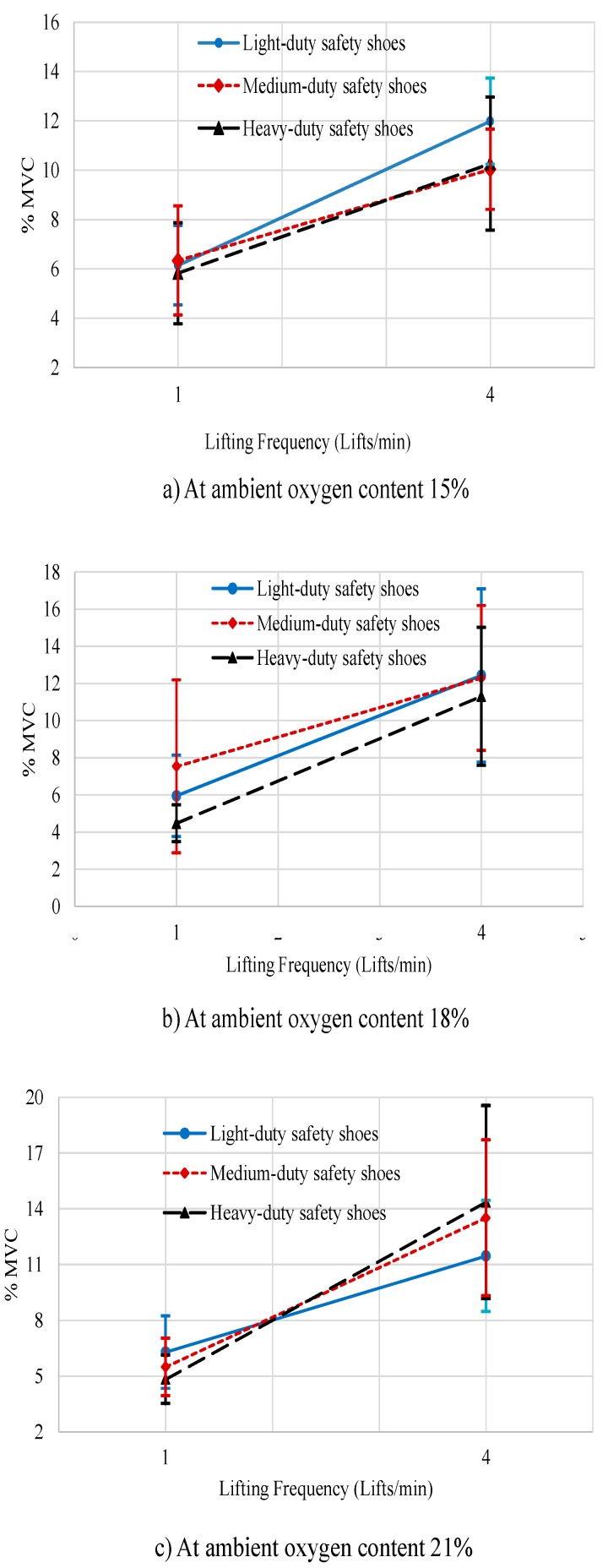
Effect of frequency of lift by ambient oxygen content by safety shoe type on %MVC of the trapezius muscle activities. (**a**) at ambient oxygen content 15%; (**b**) at ambient oxygen content 18%; (**c**) at ambient oxygen content 21%.

In general, the normalized root means square of the trapezius muscle (%MVC) at a lifting frequency of 1 lift/min was significantly lower (mean, SD = 5.871%, 2.950%) than at a lifting frequency of 4 lifts/min (mean, SD = 11.957%, 4.828%). Figure 4a has shown that for the ambient oxygen content 15%, the root mean square of the trapezius muscle (% MVC) was significantly lower when lifting frequency one lift/min while wearing the heavy safety shoes as compared to wearing of light or medium safety shoes. Furthermore, at 4 lifts/min, %MVC was significantly lower when participants wore medium safety shoes when compared to wearing light or heavy safety shoes.

Figure 4b shows that for 18% ambient oxygen content, the %MVC of the trapezius muscle was significantly lower when participants performed 4 lifts/min and wearing heavy safety shoes than when wearing the light or medium safety shoes. Similarly, the %MVC for the trapezius muscle was significantly lower when lifting frequency one lift/min when wearing heavy safety shoes than when wearing light or medium safety shoes.

Finally, at ambient oxygen content 21% (Figure 4c), the %MVC of the trapezius muscle was significantly lower when performing 4 lifts/min and wearing the light safety shoes than when wearing the heavy or medium safety shoes and the %MVC of the trapezius muscle was significantly lower when performing 1 lift/min when wearing heavy safety shoes than when wearing the light or medium safety shoes.

#### 3.3.4. Erector Spinae Muscle

The lifting frequency had significantly affected the normalized root mean square of the erector spinae muscle (%MVC), F (1.9) = 26.612, *p* < 0.001. The %MVC at a lifting frequency of 4 lift/min was significantly higher (mean, SD = 19.213%, 11.147%) than at a lifting frequency of 1 lift/min (mean (SD) = 10.703% (4.335%)).

### 3.4. Rating of Perceived Exertion (RPE)

The two main factors ambient oxygen content and lifting frequency had a significant effect on RPE (ambient oxygen content, F (2.8) = 8.484, *p* < 0.011); and lifting frequency, F (1.9) = 6.097, *p* < 0.036). The RPE was significantly less at ambient oxygen content of 18% (mean, SD = 10.5, 1.666) when compared to ambient oxygen content of 21% (mean, SD = 11.033, 1.905) or ambient oxygen content of 15% (mean, SD = 10.858, 2.015). Additionally, the RPE was significantly less when lifting frequency was 1 lift/min (mean, SD = 10.45, 1.61) when compared to 4 lifts/min (mean, SD = 11.144, 2.113).

### 3.5. Safety Shoes Discomfort Rating

The experiment showed that only the safety shoe type had a significant effect on safety shoe discomfort rating (F (2.8) = 10.721, *p* < 0.005). Safety shoe type was evaluated more comfortable when lifting at an ambient oxygen content of 21% (mean, SD = 3.86, 0.95) than when lifting at an ambient oxygen content of 18% (mean, SD = 3.82, 0.96) or at an ambient oxygen content of 15% (mean, SD = 3.66, 1.04). Furthermore, safety shoes were assessed less comfortable when lifting at frequency 1 lift/min (mean, SD = 3.67, 1.04) than lifting at frequency 4 lifts/min (mean, SD = 3.88, 0.93). The safety shoes were also assessed to be more comfortable at first replication (mean, SD = 3.79, 1.005) than at second replication (mean, SD = 3.77, 0.97). Finally, Light safety shoes were rated more comfortable during lifting (mean, SD = 4.28, 0.84) than medium safety shoes (mean, SD = 3.93, 1.06) and heavy safety shoes (mean, SD = 3.13, 1.06).

### 3.6. Rating of Oxygen Feeling (ROE)

The factor that had a significant effect on ambient oxygen rating was ambient oxygen content only F (2.8) = 6.229, *p* < 0.023. The rating at ambient oxygen content 21% (mean, SD = 4.87, 0.32) was more significant than with rating at 18% (mean, SD = 4.65, 0.73) or the rating at 15% (mean, SD = 4.30, 1.01).

## 4. Discussion

The primary purpose of this research was to study the influence of lifting in hypoxia while wearing safety shoes with the deferent frequency of lifting on the capacity of lifting, physiological responses, and stress level. The hypotheses of this study stated that oxygen content, worn safety shoes, lifting frequency, and replication have significant effect on (i) MAWL (ii) HR (beats per min), (iii) EMG of four muscle groups “trapezius, erector spinae, anterior deltoid, and biceps brachii”, (iv) rating of perceived exertion, (v) safety shoes discomfort rating, and (vi) rating of oxygen feeling. Acclimatization was provided to the participants before collecting the experimental data where previous reviews and studies [72,73,74,75] describe the changes that occur in skeletal muscle to support physical work in hypoxia. They studied a group of lowland residents at sea level, and then acclimatized them to a high altitude (5200 m). They concluded that acclimatization played an essential role in muscular work. In addition to recent research strongly emphasized the necessity of acclimatization in doing physical work [76]. The acclimatized participants’ resting heart rates were not significantly different from their parameters at pre-acclimatization sessions. In addition to acclimatization, flexibility, muscular strength, and the muscular endurance training program similar to the procedures employed by Ramadan [49] were implemented in this study to cope with the issue of specialized workers. The maximum muscular strengths of the last sequences days were not significantly different.

The findings of the study showed that the frequency of lifting decreased significantly the maximum acceptable weight of lift (MAWL), which was in harmony with the previous results described in the literature review [77]. The increasing frequency of lifting from 1 lift/min to 4 lifts/min caused a 19.8% reduction in MAWL compared to 17.1% resulted in Abadi et al. [77] and 16.67 found by Lee [47]. The reason for the differences in the amount of deficiency between the current study and previous studies was due to differences that existed in participants and the environmental conditions. In general, the MAWL decreased as the frequency of lifting increased. The MAWL in ambient oxygen content of 21% was significantly higher than those reported in ambient oxygen content of 18% by about 2.64%, as well as the MAWL in ambient oxygen content of 18% was significantly higher than those reported in ambient oxygen content of 15% by about 6.51%. The influence of decreased ambient oxygen content could be explained as increased work stress, and causing a decrease in lifting capability by 9.33% when participants transferred from 21% to 15% oxygen content room. On the other hand, the safety shoe type had no significant effect on the MAWL during work in different oxygen content environments.

The physical demand was increased- with the increase of lifting frequency and by the first replication. The results were demonstrated in terms of increase in mean heart rate. Moreover, high lifting frequency resulted in the increase if autonomic activities significantly along with the first replication. These outcomes are consistent with earlier study [12].

The biceps brachii muscle activity through performing the manual material handling tasks (lifting task) as a percent of activity for the same muscle through performing the MVC (%MVC) was highly significant when the lifting frequency was four lifts/min and decreased by 60% when the lifting frequency was one lift/min. Moreover, the biceps brachii muscle activity had low significance when wearing the heavy-duty safety shoes at low lifting frequency and was highly significant, when wearing the medium-duty safety shoes at high lifting frequency. The %MVC of anterior deltoid muscle activity was significantly higher at the high lifting frequency and decreased by 62% at the lower lifting frequency. This result was consistent with the findings of Blache’s study [78]. Additionally, the present study indicated that trapezius muscle activity %MVC was significantly low when the lifting frequency was low, which is the same when the ambient oxygen content 21% during lifting frequency was 1 lift/min and heavy-duty safety shoes were worn. In addition, the %MVC of erector spinae muscle activity was significantly lower at the low lifting frequency and increased by 79.5% at high lifting frequency, which is corroborated by the findings of Sadler, Graham, and Stevenson study [79]. The results of the muscle activities analysis indicated variable responses to the differences in workload intensity concerning the frequency of lifting and ambient oxygen content, both in combination with wearing the safety shoe type during different replications. This result suggests that different musculoskeletal system components followed different strategies in response to workload intensities, as recommended by Khalaf [80].

In this experiment, the rating of perceived exertion was highly significant with lower levels of ambient oxygen content, and higher frequency of lifting that is as indices to intensity of work; the literature supports this result for the frequency of lifting [81]. This result also agreed with the Al-Ashaik study [28], where the participants felt less perceived exertion (mean (SD), 10.45 (1.61)) in 1 lift/min when compared to their feelings (mean (SD), 11.14 (2.11)) at 4 lifts/min. Furthermore, higher scores of shoes were related to perceived higher intensity of work, appearing connotation between perceived safety shoe score and work stress. The safety shoe’s discomfort rating in this experiment props these results. The safety shoe’s discomfort rating was affected by increased intensity of workload because of decreased ambient oxygen content and an increase in the frequency of lifting. The ambient oxygen discomfort rating significantly decreased by 13.25% at ambient oxygen content 21% when compared to ambient oxygen content 15%. It was tough to compare our results regarding oxygen discomfort rating since there was no existence of a similar study available in the literature.

The results of the study suggested that guidelines must be set concerning the environmental temperature and type of safety shoes, which help in calculating the adequate weight limits. The findings could be used for updating the health and safety conditions guideline, increasing interventions in the form of education, and awareness programs, which could help reduce the medical expenditure, improve work productivity, and achieve sustainable development.

## 5. Conclusions

This paper provided evidence that ambient oxygen content increased the intensity of workload in lifting tasks. The findings show that it affected the maximum acceptable weight of the lift and the physiological responses represented in the form of heart rate and muscle activities. The results revealed that it was necessary to know the ambient oxygen content when calculating the recommended weight limits. Although lifting tasks at hypoxia was shared across various cities and countries, it was found to be located at a varying altitude of the sea level. However, none of the researchers studied the effect of oxygen on workers during manual material handling. It directs the future to examine the lifting capabilities of workers under hypoxia when wearing different types of safety shoes. Notably, it emphasized the integration of electroencephalogram (EEG), electrocardiogram (ECG), and hypoxic ventilatory response (HVR) as the dependent variables in future researches, which could help expand the research area further.

## Figures and Tables

**Table 1:** Safety shoes discomfort rating.

Description	Rating
Least comfortable	1
Less comfortable	2
Little comfortable	3
Comfortable	4
Most comfortable	5

**Table 2:** Summary of maximum acceptable weight of lift (MAWL) results.

Main Factors	Levels	Mean of MAWL (Kg)	Std. of MAWL (Kg)	*p*-Value
Ambient oxygen content	21%	17.46	3.62	0.019
	18%	17.01	3.67	
	15%	15.97	3.39	
Lifting frequency	1 lift/min	18.66	3.38	0.000
	4 lifts/min	14.96	3.73	
Replication	First	16.89	3.59	0.021
	Second	16.74	3.53	

**Table 3:** Summary of heart rate results.

Main Factors	Levels	Mean of Heart Rate (beats/min)	Std. of Heart Rate (beats/min)	*p*-Value
Lifting frequency	1 lift/min	99.98	10.52	0.000
	4 lifts/min	119.79	16.26	
Replication	First	110.85	13.36	0.021
	Second	108.92	13.42	
